# Cardioprotective mechanism of ω-3 fatty acid icosapent ethyl (IPE) in cardiomyocytes: role in high glucose and shear stress-induced mechano-transduction dysregulation

**DOI:** 10.1186/s12933-025-03033-8

**Published:** 2025-12-13

**Authors:** Ada Pesapane, Lucia Scisciola, Manuela Giovanna Basilicata, Rosaria Anna Fontanella, Nunzia Balzano, Annalisa Capuano, Asad Zia, Maryam Arshad, Zeeshan Ulfat, Giovanni Tortorella, Ludovica Vittoria Marfella, Alberta Maria Maddalena Palazzo, Giuseppe Signoriello, Celestino Sardu, Giuseppe Paolisso, Michelangela Barbieri

**Affiliations:** 1 Department of Advanced Medical and Surgical Sciences, University of Campania “Luigi Vanvitelli”, P.zza L. Miraglia, 2, 80138 Naples, Italy; 2 Department of Experimental Medicine, University of Campania “Luigi Vanvitelli”, Naples, Italy; 3 Campania Regional Centre for Pharmacovigilance and Pharmacoepidemiology, Naples, Italy; 4 Department of Mental and Physical Health and Preventive Medicine, Section of Medical Statistics, University of Campania “Luigi Vanvitelli”, Naples, Italy; 5 UniCamillus, International Medical University, Rome, Italy

**Keywords:** IPE, Omega-3 fatty acids, Hyperglycaemia, Mechano-transduction, Diabetes, Shear stress, Cardiovascular protection

## Abstract

**Background:**

Omega-3 fatty acids (FAs) are long-chain fatty acids that have shown cardioprotective effects through lipid lowering, anti-inflammatory, and membrane-stabilizing properties. In this study we investigated the molecular mechanism underlying the cardioprotective effects of icosapent ethyl (IPE), an ethyl ester of omega-3 fatty (EPA), focusing on its role on mechano-transduction, a process linking cardiac contractility to intracellular signaling, that becomes dysregulated in hyperglycaemia or disturbed blood flow, both major contributors to cardiovascular diseases.

**Methods:**

We conducted in vivo meta-analyses to assess the beneficial effects of omega-3 fatty acids on cardiac contractility and inflammation in patients with cardiovascular and cardiometabolic diseases. We investigated the effects of IPE on mechano-transduction, assessing the activation of the YAP/TAZ signalling pathway, in cardiomyocyte cells AC16 exposed to normal (NG) or high glucose (HG) conditions. We defined the role of IPE against hyperglycaemia-induced inflammation, oxidative stress, metabolism, and apoptosis by evaluating key biomarkers by Western Blot and Real-time PCR. We evaluated IPE’s impact on YAP/TAZ activation and on gene expression and protein levels of primary markers related to oxidative stress, inflammation, and metabolism in a dynamic flow model of AC16 cardiomyocytes, to mimic in vivo shear stress.

**Results:**

In vivo meta-analyses showed a significant increase of left ventricular ejection fraction (LVEF%) (mean: 0.5, 95% CI: 0.1–0.9) and a significant reduction of inflammatory markers (mean:  − 1.24, 95% CI: 2.05–0.44) in patients treated with omega-3**.** IPE treatment reduced the activation of YAP/TAZ pathway induced by HG exposure in AC16 cells. IPE partially reversed HG-induced changes in markers of inflammation, oxidative stress, metabolism and apoptosis (*p* < 0.05). Similarly, in a dynamic model of shear stress, IPE treatment mitigated the turbulent flow-mediated changes in YAP/TAZ pathway, inflammation, oxidative stress and metabolism.

**Conclusions:**

Our results demonstrate a cardioprotective role of IPE through modulation of hyperglycaemia-induced mechano-transduction dysregulation, inflammation, and oxidative stress. Additionally, our results on a shear stress model showing that IPE restores upstream regulators of YAP/TAZ and reduces disturbed flow-induced activation of pro-inflammatory pathways, suggest that IPE may exert a therapeutic effect on cardiovascular disorders associated with disturbed blood flow and hemodynamic stress.

**Graphical abstract:**

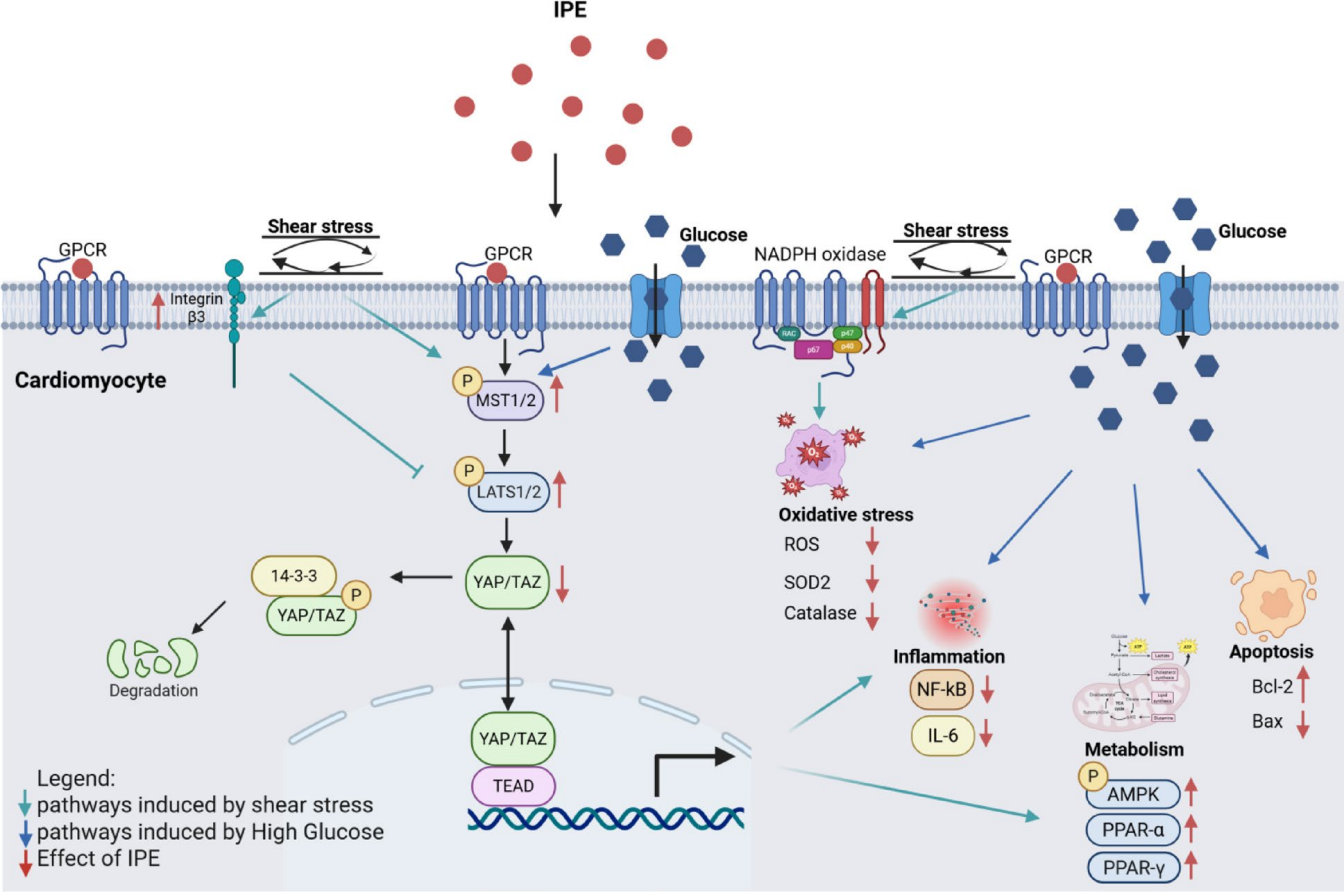

**Supplementary Information:**

The online version contains supplementary material available at 10.1186/s12933-025-03033-8.

## Research Insights

**What is currently known about this topic ?**

- Omega-3 fatty acids improve lipid profiles and lower pro-inflammatory cytokines. Omega-3 reduces cardiovascular risk in T2DM patients by improving cardiometabolic biomarkers.

**What is the key research question ?**

- Does ω-3 fatty acid icosapent ethyl (IPE) exert a cardioprotective role in high glucose and shear stress-induced mechano-transduction impairment ?

**What is new ?**

- We studied IPE effects in cardiomyocytes under shear stress as a model of hemodynamic cues. IPE exerted a cardioprotective role by modulating mechano-transduction in hyperglycaemia. IPE exert a beneficial effect on inflammation and oxidative stress, supporting its beneficial effects on cardiomyocytes.

**How might this study influence clinical practice ?**

- Findings suggested IPE therapeutic potential for mechano-transduction-related diseases.

## Introduction

Omega-3 fatty acids (FAs) are long-chain fatty acids that reduce triglycerides (TG), downregulate mechanisms of atherosclerosis and reduce cardiovascular events [1, 2]. Omega-3 fatty acids have been shown to improve cardiometabolic biomarkers in patients with type 2 diabetes mellitus (T2DM), producing beneficial effects on lipid profiles, reducing pro-inflammatory cytokines and enhancing glycaemic control, thereby contributing to a reduced cardiovascular risk and mortality [3, 4].

Icosapent ethyl (IPE), the ethyl ester of eicosapentaenoic acid (EPA), exerts multiple cardiovascular benefits: it lowers TG , modulates inflammation, possesses antioxidant properties, and contributes to atherosclerotic plaque stabilization [5, 6].

The REDUCE-IT trial demonstrated significant cardiovascular mortality benefits of IPE, with a 25% relative risk reduction of major cardiovascular events [5, 7]. The EVAPORATE study provided strong evidence of the beneficial clinical effects of IPE on the progression of atherosclerosis process [8].

Hyperglycaemia, one of the most relevant cardiovascular risk factors, has been shown to interfere with mechano-transduction, the process that links cardiac contraction to intracellular signaling, through which cardiomyocytes convert mechanical stimuli into cellular responses, resulting in activation of signalling pathways and transcriptional regulation [9–12]. Under hyperglycemic stress, YAP and TAZ, central regulators of mechano-transduction signaling and downstream effectors of the Hippo pathway [13, 14], are activated and translocate into the nucleus, where they promote the transcription of pro-inflammatory and pro-atherogenic genes [15].

Notably, YAP/TAZ respond not only to metabolic alterations but also to mechanical stimuli [16], and function as a central signalling node integrating both types of cues relevant to cardiomyocyte dysfunction, therefore representing a suitable mechanistic target to evaluate how these stimuli modulate inflammatory and oxidative stress responses.

Shear stress represents an important determinant of the cardiac mechanical environment. In the ventricles, blood flow generates endocardial shear stress with high spatial and temporal heterogeneity, exposing endocardial endothelium and subendocardial cardiomyocytes to complex mechanical forces that influence cardiac function and remodelling [17, 18].

Cardiomyocytes sense and respond to the mechanical forces generated by blood flow, such as cyclic stretch, wall stress, and load variations, as well as to paracrine signals by shear stress induced endocardial cells [19, 20].

Alterations in vascular shear stress may also affect cardiomyocyte function indirectly, through microvascular dysfunction and damage to the endothelial glycocalyx [21]. Such alterations can impair oxygen delivery and endothelial function, especially in conditions like hyperglycaemia or microangiopathies, where the crosstalk between the endothelium and cardiomyocytes becomes particularly relevant [22, 23].

To get results on the cardioprotective mechanisms of omega-3 fatty acid IPE in cardiomyocytes, we firstly performed in vivo meta-analyses to assess the beneficial effects of omega-3 fatty acids on cardiac contraction and inflammation in patients with cardiovascular and cardiometabolic diseases.

Then we focused on the cardioprotective mechanism of omega-3 fatty acid IPE in an in vitro model of cardiomyocytes, to investigate whether IPE can reduce the harmful effects of high glucose on mechano-transduction signalling, as well as on inflammation, oxidative stress, metabolism and apoptosis. Moreover, the effects of IPE in cardiomyocytes exposed to shear stress, used as an experimental model of hemodynamic stimulation, were examined to characterize their responses to mechanical forces and to identify new potential therapeutic strategies targeting diseases associated with altered mechano-transduction.

## Methods

### Meta-analysis: search strategy, selection criteria, endpoint, and statistical analysis

A systematic literature review was conducted by searching the PubMed database for randomized clinical trials from 2006 to 2024.

For the meta-analysis on Left ventricular ejection fraction (LVEF), the review included patients diagnosed with cardiovascular diseases (162 with chronic heart failure and 358 with acute myocardial infarction (MI) treated with omega-3 fatty acids or placebo. Only studies in which omega-3 fatty acids were the sole investigational intervention, without additional supplements or experimental treatments except for clinically indicated medications, were considered. Studies were excluded if they lacked a control or placebo group, combined omega-3 with other experimental interventions or supplements, enrolled non-adult populations or patients without documented cardiovascular disease, or did not provide sufficient data for pooling.

Data from published reports and previous meta-analyses were utilized, and all included manuscripts were manually searched for any additional studies. Among the studies evaluated, three were found to be eligible, while others were excluded for not meeting eligibility criteria. Data from 358 patients included in the OMEGA-REMODELED STUDY [24], 133 in the study by Nodari et al. [25], and 29 in the randomized pilot trial by Moertl et al. [26] were pooled for the meta-analysis, analyzing 260 patients treated with OMEGA-3 and 260 control patients (placebo). The primary outcome assessed was change in the % of LVEF. Results are expressed as mean % change + SD. A random-effects meta-analysis was conducted, and the index of study heterogeneity (I2 = 69.84%) indicated high heterogeneity. A forest plot of the meta-analysis was created using Stata software (version 16.0, Stata Corp., College Station, TX).

For the meta-analysis on inflammatory markers, the review included patients diagnosed with cardiometabolic diseases (heart failure (HF), cardiovascular diseases (CVDs), T2DM). Data from published reports and previous meta-analyses were utilized, and all included manuscripts were manually searched for any additional studies. Only studies investigating omega-3 fatty acids as the exclusive experimental treatment, without additional supplements or other interventions except for clinically necessary medications, were included. Eligible studies were required to report quantitative data on inflammatory markers, including IL-6, TNF-α, or CRP. Studies were excluded if they lacked a control or placebo group, combined omega-3 with other experimental interventions or supplements beyond clinically necessary medications, enrolled non-adult populations or patients without well-documented cardiometabolic diseases, CVDs or T2DM.

Data from 607 patients from 2 studies for CVDs [27, 28], 3 studies for T2DM [29, 30], 6 studies for HF [25, 26] [31, 32] were pooled for the meta-analysis, analyzing 312 patients treated with OMEGA-3 and 295 control patients (placebo). The primary outcome assessed was expression of TNF-α, IL-6 and CRP. Results are expressed mean + SD. A random-effects meta-analysis was conducted, and the index of study heterogeneity (I2 = 96.56%) indicated high heterogeneity. A forest plot of the meta-analysis was created using Stata software (version 16.0, Stata Corp., College Station, TX).

PRISMA flow charts describing the procedure to select studies to include in the meta-analyses were provided.

To assess the influence of each individual study on the overall effect of the meta-analysis, a sensitivity analysis (Leave-one-out analysis) was performed using Stata 18.

### Cell culture

The human cardiomyocyte cell line AC16 was purchased from EMD Millipore (cod. SCC109). Cells were cultured in Dulbecco’s Modifed Eagle’s Medium (DMEM)/F12 (cod. AL215A, Microgem) containing 12.5% fetal bovine serum (FBS) (cod. ECS0180L, Euroclone), 1% antibiotics penicillin–streptomycin (cod. ECB3001D, Euroclone), and 1% of L-glutamine (cod. ECB3000D, Euroclone) and maintained in a humidified incubator at 37 °C and 5% CO_2_. The cells were grown between 5 and 7 passages, and experiments were performed in triplicate. AC16 cells were exposed to normal (NG) and high glucose (HG) 33 mmol/L D glucose (cod. G8644, EMD Millipore) for 7 days and treated with Icosapent ethyl (IPE) (cod. Amb10843525, Ambinter c/o Greenpharma) at a concentration of 40 µM. The medium was changed every 48 h. Normal glucose (NG), considered the control, are cells exposed to normal glucose concentration (5.5 mmol/L) and cultured for 7 days. The concentration of IPE to carry out experiments was determined by a dose–response curve, using cell viability assay. Mycoplasma testing was conducted before use.

### Dynamic cell cultures

The cardiomyocytes cell line AC16 was additionally exposed to fluidic conditions. Briefly, AC16 cells (1 × 10^4^ cells) were seeded onto glasses discs (Ivtech) assembled in the bottom of a flow chamber (LB1, Ivtech), a single flow transparent bioreactor with an internal hydrodynamic design which ensures a wall shear stress for high fluid turnover rates. The LB1 chambers were connected by silicon tubes to an Ivtech Pump System (Ivtech), a peristaltic pump, which creates the flow. Cells were perfused in the LiveBox1 chamber at flow rates of 500 µL/min with DMEM/F12 with 12.5% FBS (normal concentration of glucose), in presence or absence of IPE at 40 µM for 72 h. The increase in flow, together with the chamber configuration, leads to a turbulent flow regime above a certain flow rate (e.g., from 300 µL/min). Shear stress is not uniform, but shows different values at different points within the chamber, with a maximum value in the central region of the glass and decreasing values in the region close to the borders. Shear stress in this system can’t be expressed as a single value, as this would not be statistical significant. Further details regarding the use of blood flow unit, expressed as µL/min, are reported in Supplementary Fig. 1. The flow system was maintained at 37 °C, and the circulating medium was equilibrated with a humidified atmosphere of 5% CO_2_. After the experiment, when the cells reached a confluence of 80%, the LB1 chambers were disassembled and the glass discs removed from the bottom part and used for further investigations and analysis.

### Protein extraction and western blot analysis

Proteins were extracted in a lysis buffer containing protease inhibitors (10 mM Tris HCL pH8, 150 mM NaCl, 10 mM NaF, 1% NP40, 1 mM PMSF). Then, 30 µg of proteins were subjected to 10% sodium dodecyl sulfate–polyacrylamide gel electrophoresis (SDS-PAGE) and transferred to 0.22 µm polyvinylidene fluoride (PVDF) membranes. Membranes were blocked with 5% milk in TBS-T (Tween 20 buffered with 0.15% Tris pH 8) at room temperature for 1 h and then incubated with the following primary antibodies diluted in TBS-T: p-MST1 (1:1000) (#49,332, Cell signaling Technology), p-LATS1 (1:1000) (#8654, Cell signaling Technology), active YAP (1:1000) (#ab205270, Abcam), TAZ (1:1000) (#83,669, Cell signaling Technology), Oxidative stress defense western blot cocktail (1:1000) (# ab179843 Catalase, SOD1, TRX, Abcam), p-NF-kB (1:1000) (E-AB-68092, ELAB Science), IL-6 (1:1000) (E-AB-30095, Elabscience), SOD2 (1:1000) (#ab13533, Abcam), p-AMPK (1:1000) (E-AB-21121, ELAB Science), PPAR-α (1:1000) (#ab227074, Abcam), PPAR-γ (1:1000) (#2443, Cell signaling Technology), Integrin β3 (1:1000) (#13,166, Cell signaling Technology), Bcl-2 (1:1000) (E-AB-15522, ELAB Science), Bax (1:1000) (E-AB-22128, ELAB Science) overnight at 4°C. Vinculin (1:10,000) (#ab129002, Abcam) or tubulin (1:1000) (#5568, Cell signaling Technology) were used for normalization of protein expression as internal controls. After three washes in TBS-T, the membrane was incubated with the corresponding secondary antibodies, goat anti-rabbit IgG-h + HRP conjugated (1:10,000) (cat. A120-101P, Bethyl), for 1 h at room temperature. Immune complexes were visualized using Clarity Max Western ECL substrate (cat. 1,705,062, Bio-Rad Laboratories) and visualized using the ChemiDoc imaging system with Image Lab Software version 6.1 (Bio-Rad Laboratories). The molecular weight of the proteins was estimated with pre-stained protein markers (cat. G623 Opti-Protein-Marker, ABM). Densitometric analysis was performed using Image J software.

### RNA extraction and real time PCR

Total RNA was isolated and purified using miRNeasy Mini Kit (cat. 217,004, Qiagen) according to the manufacturer’s instructions. Then cDNA was synthesized from 1 µg of total RNA using QuantiTect Reverse Transcription Kit (cat. 205,310, Qiagen). mRNA levels were determined by qPCR with Green-2 Go qPCR master mix (# QPCR004-5 Biobasic) using Rotor-GENE Q (Qiagen).

Primer’s sequence: NF-κB: fw 5′-AATGGTGGAGTCTGGGAAGG-3′, rv 3′-TCTGAC GTTTCCTCTGCACT-5′; SOD2: fw 5'-AAGTCATCCACCCACCTCAG-3', rv 5'-CGTGGAGAGAGGCATGAAAGC-3'; IL-6: fw 5'-AGTCCTGATCCAGTTCCTGC-3', rv 5'- CTACATTTGCCGAAGAGCCC-3'; PPAR-α: fw 5’- CAGGCTATCATTACGGAGTCC-3’, rv 5’-TTCTGTTCTTTTTCTGGATCTTGC-3’; PPAR-γ: fw 5’- GTTTCAGAAATGCCTTGCAGT-3’, rv 5’-GGATTCAGCTGGTCGATATCAC-3’; Bcl-2: fw 5′-GATGACTGAGTACCTGAACCG-3′, rv 5′AGCCAGGAGAAATCAAACAGAG-3′, Bax: fw 5′-CAAACTGGTGCTCAAGGC-3′, rv 5′-AAAGATGGTCACGGTCCAAC-3′; β-actin: fw 5′-CATCCGCAAAGACCTGTACG-3′, rv 5′-CCTGCTTGC TGATCCACATC-3′.

For each amplification cycle a threshold cycle value (Ct) was obtained and the ΔCt was calculated as the difference in Ct between the target mRNA and the housekeeping mRNA (β-actin). The increase in mRNA expression compared to NG was calculated using the 2^−ΔΔCt^ method.

### Immunofluorescence

AC16 cells (number = 1 × 10^4^) were plated in the LB1 chambers (Ivtech), connected to the peristaltic pump (Ivtech), which creates the flow. Cells were perfused in the LiveBox1 chamber at flow rates of 500 µL/min with DMEM/F12 with 12.5% FBS (normal concentration of glucose), in presence or absence of IPE at 40 µM for 72 h (80% confluency) to simulate a turbulent flow. The same number of cells plated in static model in DMEM/F12 with 12.5% FBS (normal concentration of glucose) were used as negative control. Then, the medium was removed and the cells were washed two times in PBS. The cells were then fixed in formaldehyde 4% for 15 min at RT, washed 3 times in PBS and permeabilized in PBS-TritonX-100 (0.1%) for 20 min at RT. After blocking cells with 10% BSA in PBS for 1 h at RT, the cells were incubated overnight with 1°AB active YAP (#ab205270, Abcam), diluted (1:500) in 1%BSA-PBS at 4 °C. The following day, after 3–5 times with PBS, a fluorescent Alexa Fluor 488 Goat Anti-Rabbit IgG (#ab150077, Abcam) diluted in 1% BSA-PBS (1:1000) was added as secondary antibody for 1 h at RT. DAPI was used as a nuclear counter stain. Slides were mounted with 50% glycerol in PBS. Slides were imaged with a Leica Thunder Imager 3D assay Fluorescent microscopy. The analysis was performed by ImageJ software.

### ROS detection

Intracellular ROS levels were measured using a ROS detection assay kit (#ab113851 DCFDA / H2DCFDA—Cellular ROS Assay Kit, Abcam).

Cells were cultured in cell culture media in the presence of HG and IPE so that 3 × 10^6^−4 × 10^6^ cells are obtained the day before the experiment. According to the manufacturer’s instructions, the cells were harvested and seeded in a dark, clear bottom 96-well microplate at 25,000 cells/well and allowed to adhere overnight. The cells were then washed with 1X wash buffer and stained by adding 100 µL/well of the diluted DCFDA Solution for 1 h at 37 °C in the dark. DCFDA Solution was then removed and the cells were washed in 1X wash Buffer. Fluorescence was measured on a fluorescence microplate reader (Sunrise absorbance reader, Techan) at Ex/Em = 485/535 nm. Blank readings were subtracted from all measurements and fold change was determined from assay control.

### Statistical analysis

All experiments were performed with three independent biological replicates. Results are expressed as the mean ± SEM. All data were assessed for normality using the Shapiro–Wilk test, which confirmed that the data were normally distributed. The differences between the mean values were assessed by a one-way analysis of variance (ANOVA) test. Differences were considered significant at a *p*-value of < 0.05. The data were analysed by SPSS IBM Version 23. GraphPad Prism software was used for drawing figures.

## Results

### Beneficial effects of omega-3 fatty acids on cardiac contraction and inflammation in vivo

To assess in vivo the beneficial effect of omega-3 fatty acids on cardiac contraction, the LVEF% by a random-effect meta-analysis of patients diagnosed with cardiovascular diseases (162 with chronic heart failure and 358 with acute MI) treated with omega-3 fatty acids or placebo was evaluated. A PRISMA flow chart describing the selection process for the included studies in the meta-analysis was provided (Supplementary Fig. 2).

**Fig. 1:**
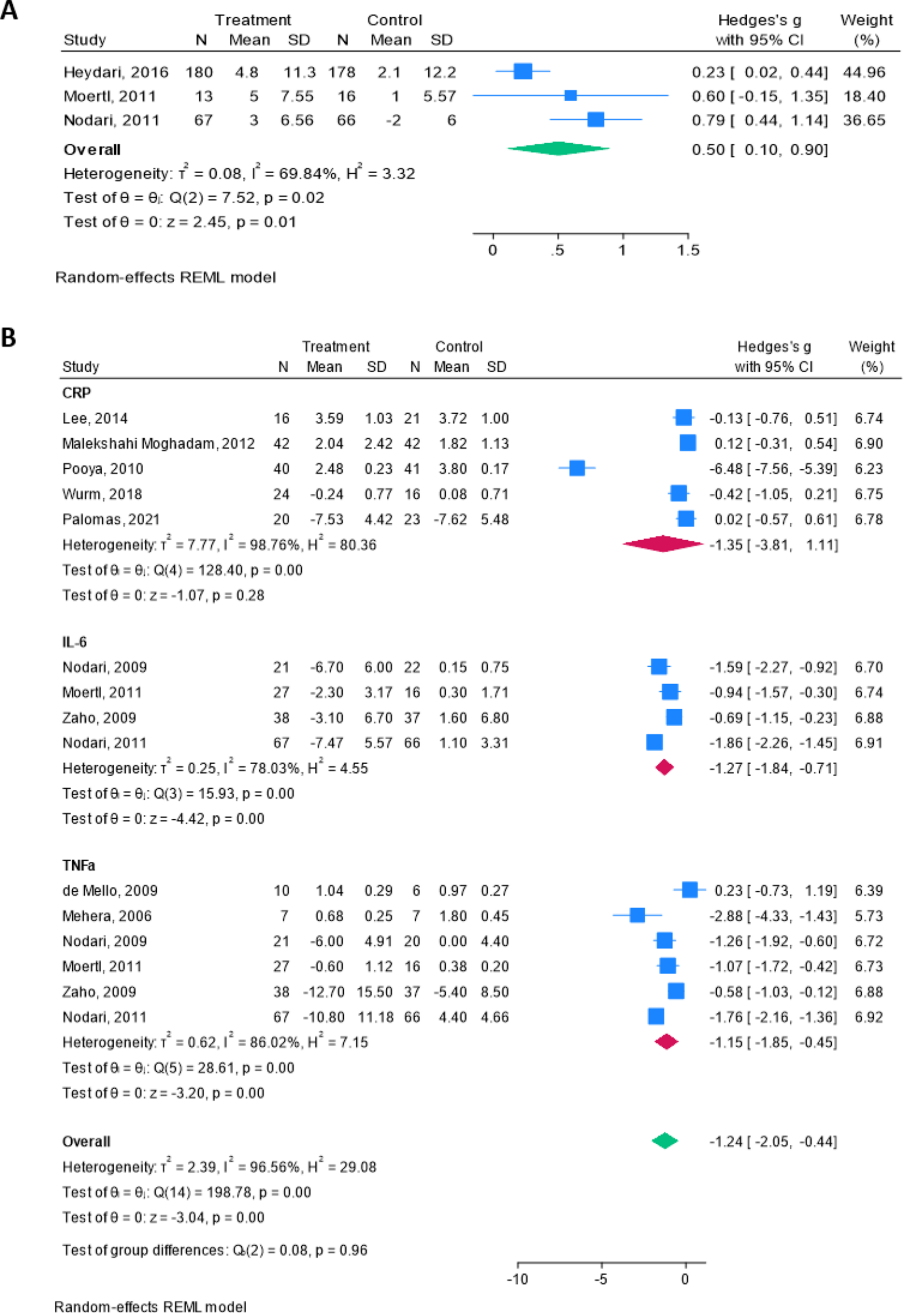
Meta-analysis of beneficial effects of omega-3 fatty acids in patients with Cardiovascular and cardiometabolic diseases. **A** Forest plot of CVD patients treated with omega-3 fatty acids or placebo. Data are shown as mean change + SD. The graph was created using Stata software (version 16.0, Stata Corp., College Station, TX). **B** Forest plot of inflammation markers (IL-6, TNF-α, CRP) in patients with cardiometabolic diseases treated with omega-3 fatty acids or placebo. Data are shown as Mean + SD. The graph was created using Stata software (version 16.0, Stata Corp., College Station, TX)

The overall results showed a mean change of 0.59 (95% CI 0.10–0.90, Heterogeneity: r^2^ = 0.08, I^2^ = 69.84%, H^2^ = 3.32), indicating that omega-3 treatment has a protective effect on LVEF% compared to control (Fig. 1A).

In a further random-effect meta-analysis, the anti-inflammatory effects of omega-3 fatty acids in vivo in patients with cardiometabolic diseases were also investigated. A PRISMA flow chart describing the selection process for the included studies in the meta-analysis was provided (Supplementary Fig. 3). So far, 607 patients diagnosed with cardiometabolic diseases (Fig. 1B) treated with omega-3 fatty acids or placebo for the expression of the main inflammation markers: TNF-α, IL-6 and CRP, were analyzed.

IL-6 and TNF-α were significantly reduced in the omega-3 treated patients, with mean changes and 95% confidence intervals of − 1.27 (− 1.84, − 0.45) and − 1.15 (95%CI = − 1.85, − 0.45), respectively. CRP was reduced with mean change and 95% CI of − 1.35 (95%CI = − 3.81, 1.11), but the differences are not significant, due to high heterogeneity of the studies (I^2^ = 98.8%). Overall results showed a significant decrease of the analysed inflammatory markers in patients treated with Omega-3 fatty acids with a mean change of − 1.24 and 95%CI = − 2.05, − 0.44 (*P* < 0.01), despite the high heterogeneity (I^2^ = 96.6%), demonstrating their efficacy in improving patients inflammatory profile (Fig. 1B).

To evaluate the influence of individual studies on the meta-analytic results, a leave-one-out sensitivity analysis was conducted. For TNF-α and IL-6, the pooled effects remained stable, with minimal variation across iterations; despite high heterogeneity, the overall estimates were not compromised. In contrast, the CRP endpoint showed marked instability, with one or two influential studies driving substantial shifts in the pooled effect and further increasing heterogeneity (Supplementary Fig. 4).

### Effects of IPE on mechano-transduction in a static model of cardiomyocytes exposed to HG

**Fig. 2:**
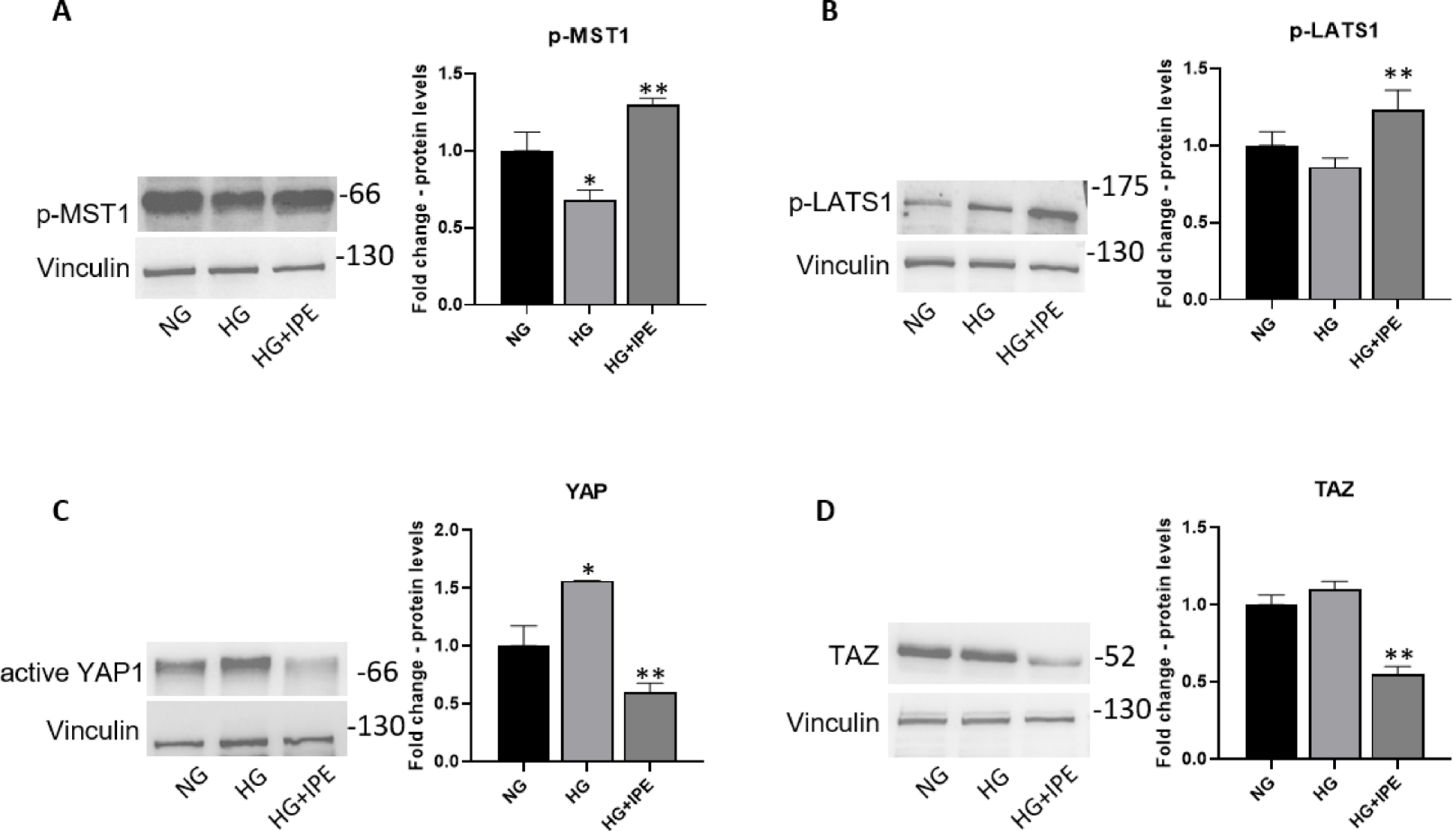
IPE effects on mechano-transduction in AC16 cells exposed to hyperglycemia. Western blot analysis for p-MST1 (**A**), p-LATS1 (**B**), active YAP (**C**) and TAZ (**D**) in AC16 cells exposed to NG concentration (NG), cells exposed to high glucose concentration (HG), and cells co-treated with HG and 40 µM IPE (HG + IPE). The histograms show the densitometric analysis of 3 separate experiments representing the relative expression of proteins; NG value was set as 1. Data are mean ± SEM. * *P* < 0.05 versus NG; ** *P* < 0.05 versus HG 7 days

The activation of the YAP/TAZ pathway for mechano-transduction by the analysis of the protein expression levels of the main signaling pathway mediators was analysed in cardiomyocytes in high glucose conditions in absence and presence of IPE. The concentration of IPE to use in the experiments was determined by a dose-curve response (Supplementary Fig. 5). HG treatment for 7 days induced a significant reduction in protein expression levels of p-MST1, a kinase upstream to YAP/TAZ, compared to normal glucose condition in a static model of AC16 cardiomyocytes (0.68-fold vs NG, *p* < 0.05); IPE treatment reverted such HG-induced effect (1.91-fold vs HG; *p* < 0.05) (Fig. 2A). Moreover, exposure to HG induced a decrease in protein expression levels of p-LATS1, a kinase upstream to YAP/TAZ and downstream to p-MST1, and this effect was reverted by IPE addition (1.43-fold change vs HG; *p* < 0.05) (Fig. 2B).

HG treatment for 7 days induced a significant increase in active YAP, downstream effector of the Hippo pathway, in cardiomyocytes AC16 cells compared to normal glucose condition in a static model (1.5-fold change vs NG; *p* < 0.05); co-treatment with IPE reduced such HG-mediated impact (0.4-fold change vs HG; *p* < 0.05) (Fig. 2C). Similarly, HG induced an increase in TAZ protein expression levels. In contrast, the Ac16 cells exposed to HG and treated with IPE showed that IPE antagonized such HG-induced upregulation (0.5-fold change vs HG; *p* < 0.05) (Fig. 2D).

### Effects of IPE on inflammation and oxidative stress in cardiomyocytes exposed to HG

**Fig. 3:**
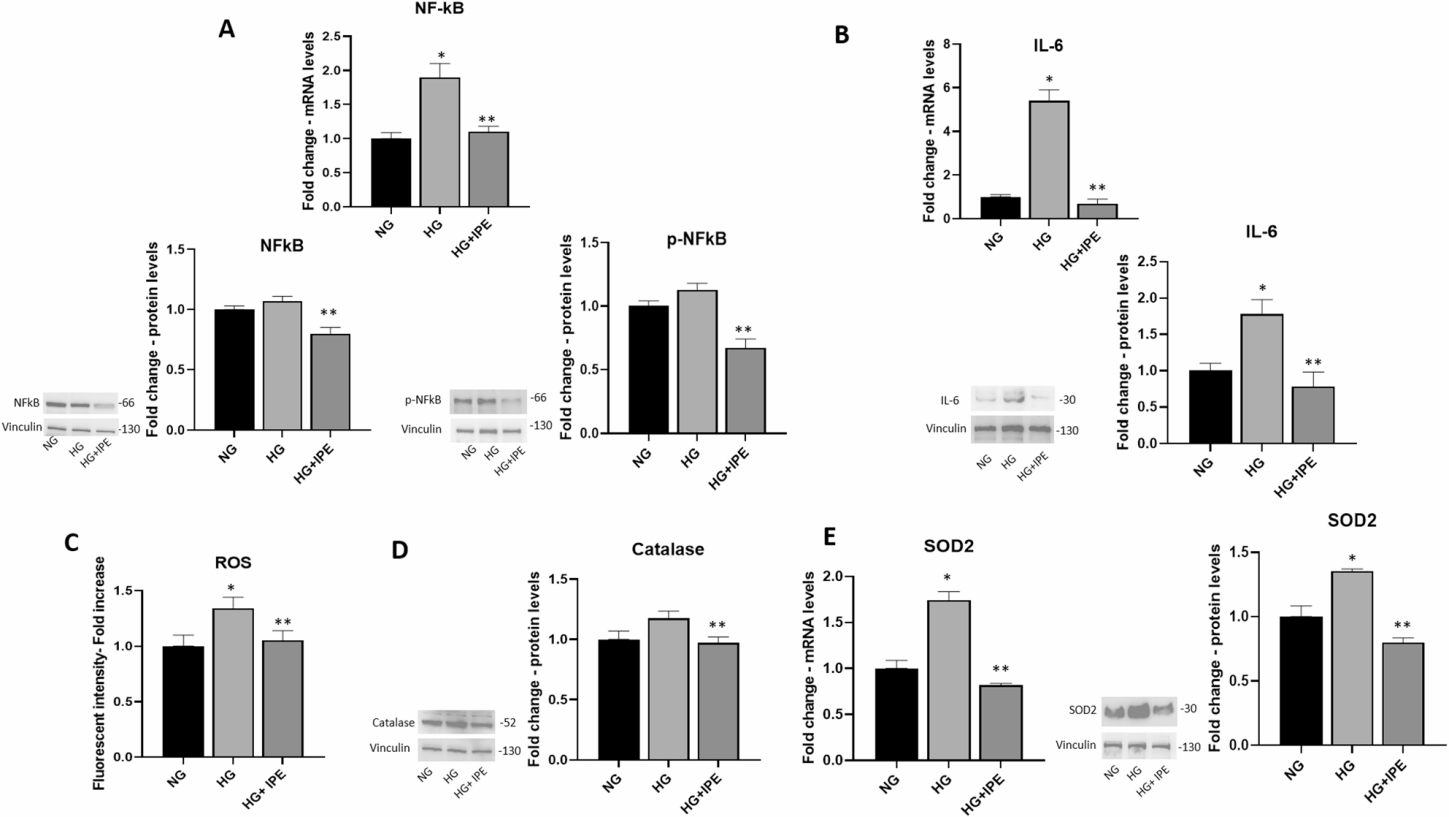
Effects of IPE on inflammation and oxidative stress in response to HG treatment

Treatment with HG for 7 days induced a significant increase of NF-kB mRNA expression levels in AC16,and an increase of protein expression levels for NF-kB and for p-NF-kB (Ser276), marker of NF-kB activity. Co-treatment with IPE prevented such HG-induced changes for this inflammatory marker (*p* < 0.05 vs HG) (0.59-fold change vs HG; *p* < 0.05) (Fig. 3A).

HG also induced a significant increase of mRNA and protein expression levels for IL-6; IPE was able to prevent such negative HG-mediated effect (0.43-fold change vs HG; *p* < 0.05) (Fig. 3B).

Measurement of ROS levels, as a biomarker of oxidative stress, was also investigated. HG treatment for 7 days induced a significant increase in ROS levels in AC16 cells (1.34-fold change vs NG; *p* < 0.05). Co-treatment with IPE countered such HG-mediated impact in this cellular model (0.75-fold change vs HG; *p* < 0.05) (Fig. 3C).

mRNA levels and protein concentration of the main genes involved in oxidative stress were also quantified. HG induced an increase in the protein expression levels of catalase, a protein involved in the protection against oxidative stress, which is significantly reduced in response to IPE treatment (*p* < 0.05 vs HG) (Fig. 3D). Moreover, HG treatment induced an increment in the mRNA and protein expression levels of SOD2, a potent antioxidant enzyme that acts as a ROS scavenger, in cardiomyocytes AC16 (1.35-fold change vs NG; *p* < 0.05). A statistically significant reduction in the expression levels of this inflammatory marker was observed in cells co-treated with IPE, at both mRNA and protein expression (0.58-fold change vs HG; *p* < 0.05) (Fig. 3E).

### Effects of IPE on metabolism in AC16 cardiomyocytes in response to HG

The impact of IPE on metabolism changes was also investigated in AC16 cardiomyocytes cell line exposed to hyperglycaemia.

**Fig. 4:**
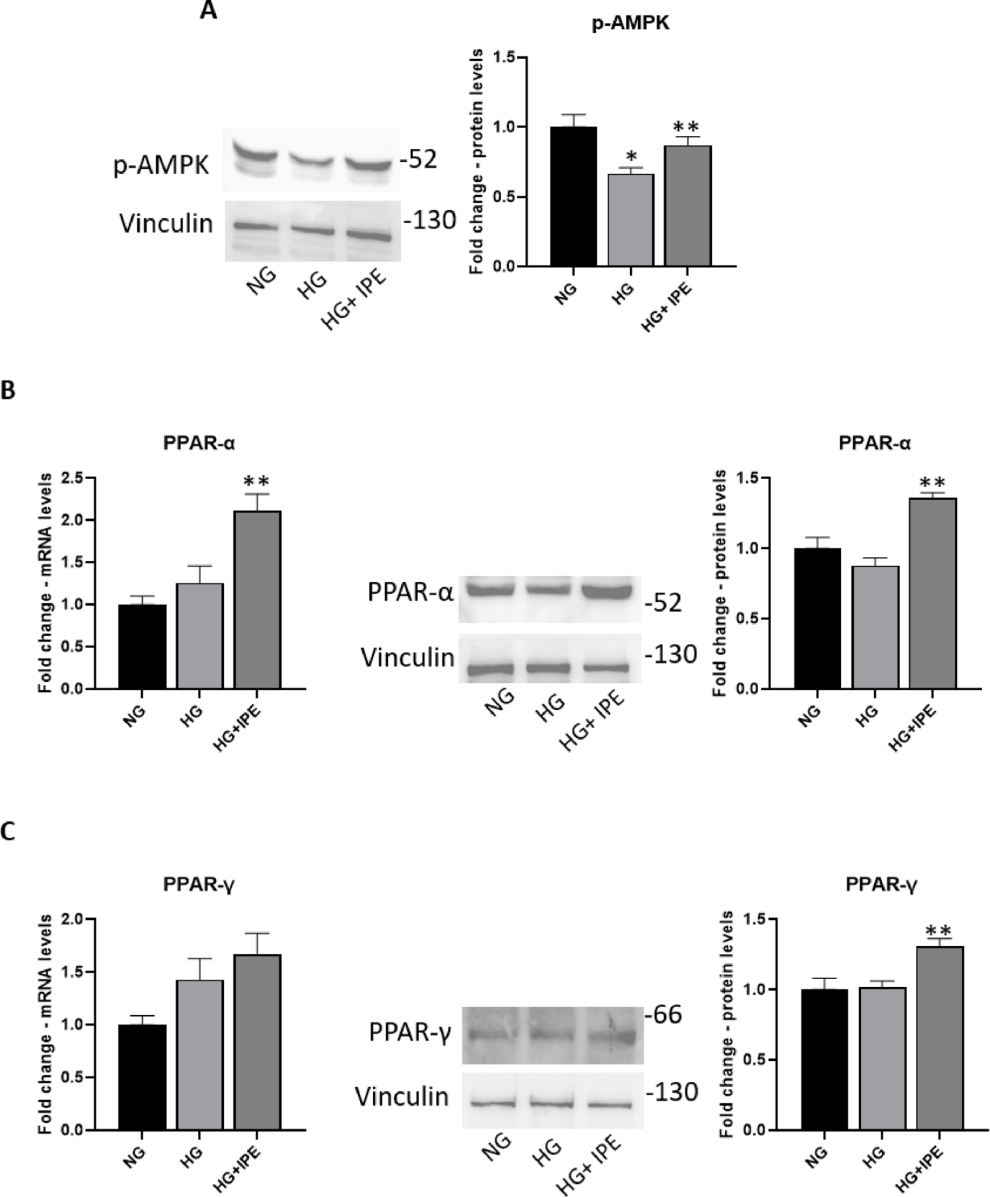
IPE effects on metabolism in a static model of cardiomyocytes exposed to HG. Western blot for p-AMPK (**A**), PPAR-α (**B**, right panel) and PPAR-γ (**C**, right panel) in AC16 exposed to NG, HG, HG + IPE. The histograms show the densitometric analysis of 3 separate experiments representing the relative expression; NG value was set as 1. Data are mean ± SEM. * *P* < 0.05 versus NG; ** *P* < 0.05 versus HG 7 days. qRT-PCR for PPAR-α (**B**, left panel) and PPAR-γ (**C**, left panel) in AC16 exposed to NG, HG, HG + IPE. β-Actin was used as internal control. The fold increase of mRNA expression compared with NG was calculated using the 2^−ΔΔCt^ method. Data are mean ± SEM. * *P* < 0.05 vs NG; ** *P* < 0.05 vs HG 7 days

HG treatment induced a significant reduction of protein expression levels for p-AMPK in AC16 (0.66-fold change vs NG; *p* < 0.05); IPE is able to prevent such HG-induced effect (1.32-fold change vs HG; *p* < 0.05) (Fig. 4A).

Treatment with HG for 7 days did not induce changes of mRNA expression and protein levels for PPAR-α in AC16; the addition of IPE induced a significant increase in both mRNA expression (1.66-fold change vs HG; *p* < 0.05) and protein levels (1.55-fold change vs HG; *p* < 0.05) for PPAR-α (Fig. 4B). In a similar manner, the addition of IPE induced an increase in both mRNA expression and protein levels (1.3-fold change vs HG; *p* < 0.05) for PPAR-γ, while the treatment with only HG for 7 days did not change its mRNA expression and protein levels (Fig. 4C).

### Effects of IPE on apoptosis in cardiomyocytes AC16 exposed to HG

**Fig. 5:**
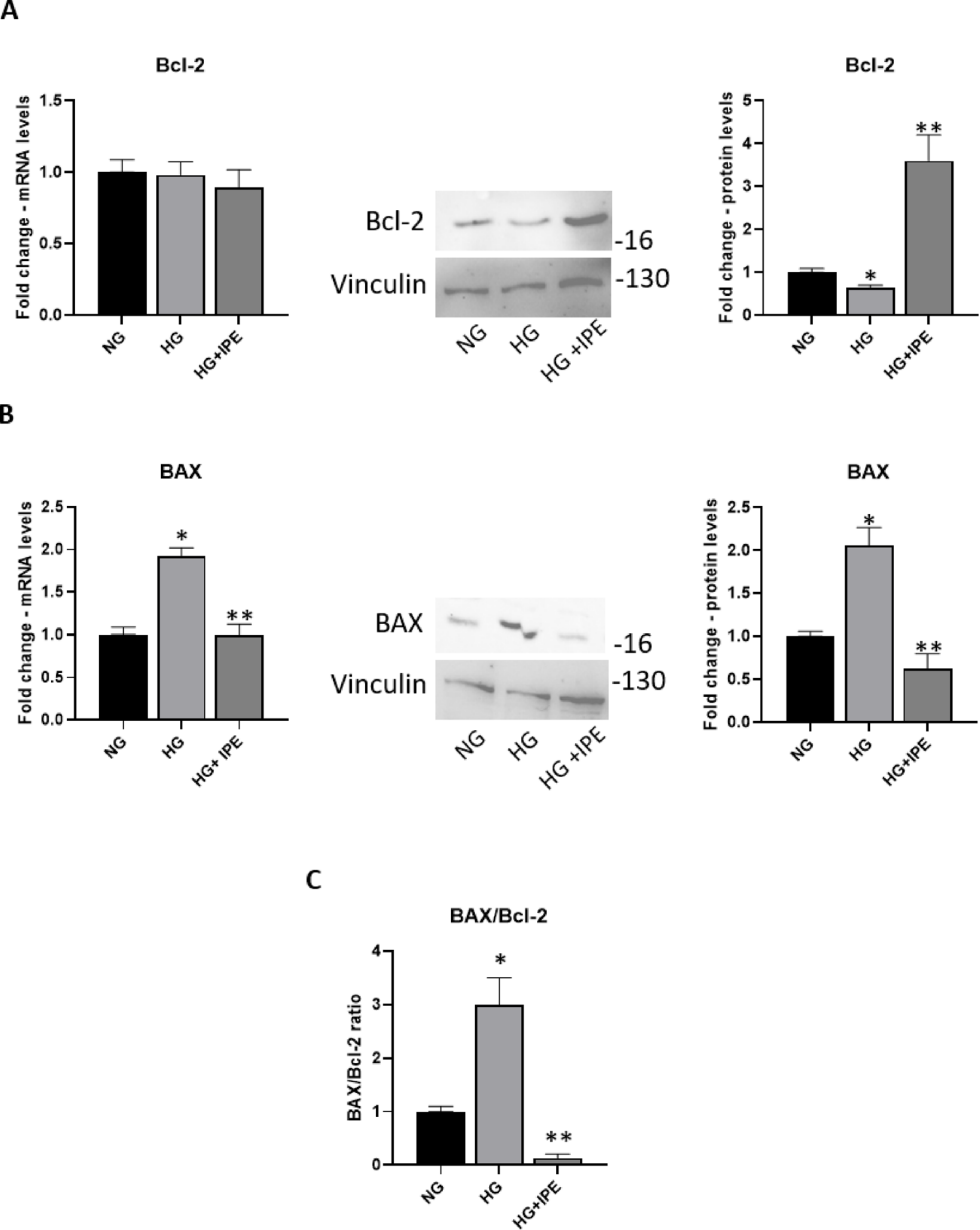
Effects of IPE on apoptosis in cardiomyocytes exposed to hyperglycemia: **A**, **B** Bcl-2 and Bax mRNA and protein expression levels in AC16 cells exposed to NG, HG, HG with 40 µM of IPE. For q-RT PCR, β-Actin was used as internal control. The fold increase of mRNA expression compared with NG was calculated using the 2^−ΔΔCt^ method. Data are mean ± SEM. * *P* < 0.05 vs NG; ** *P* < 0.05 versus HG 7 days. For Western blot, the histograms show the densitometric analysis of 3 separate experiments representing the relative expression being NG value set as 1. **C** BAX/Bcl-2 protein expression ratio in AC16 in response to NG, HG, HG + IPE. Data are mean ± SEM. *p < 0.05 versus NG; **p < 0.05 versus HG 7 days

7 days HG treatment induced a significant reduction in the protein expression levels of the anti-apoptotic protein Bcl-2 (0.64-fold change vs HG; *p* < 0.05), whereas no changes in mRNA expression were observed. IPE treatment prevented the HG-induced down-regulation of Bcl-2 protein levels (5.63-fold change vs HG; *p* < 0.05) (Fig. 5A).

HG treatment significantly increased Bax mRNA (1.9-fold change vs NG; *p* < 0.05) and protein levels (2.06-fold change vs HG; *p* < 0.05) in AC16 cells; the addition of IPE prevented the mRNA (0.47-fold change vs HG; *p* < 0.05) and protein increase (0.47-fold change vs HG; *p* < 0.05) (Fig. 5B).

Moreover, the BAX/Bcl-2 protein ratio, a relevant apoptotic marker, was significantly increased by HG treatment in cardiomyocytes AC16 cells (threefold change vs NG; *p* < 0.05); IPE reduced such HG-mediated impact on this apoptotic index (0.043-fold change vs HG; *p* < 0.05) (Fig. 5C).

### Effects of IPE on mechano-transduction in a dynamic shear stress model of cardiomyocytes

IPE effects on mechano-transduction were also investigated in a dynamic model of shear stress, exposing cardiomyocytes to turbulent flow in a normal glucose condition and analyzing the activation of YAP/TAZ pathway.

**Fig. 6:**
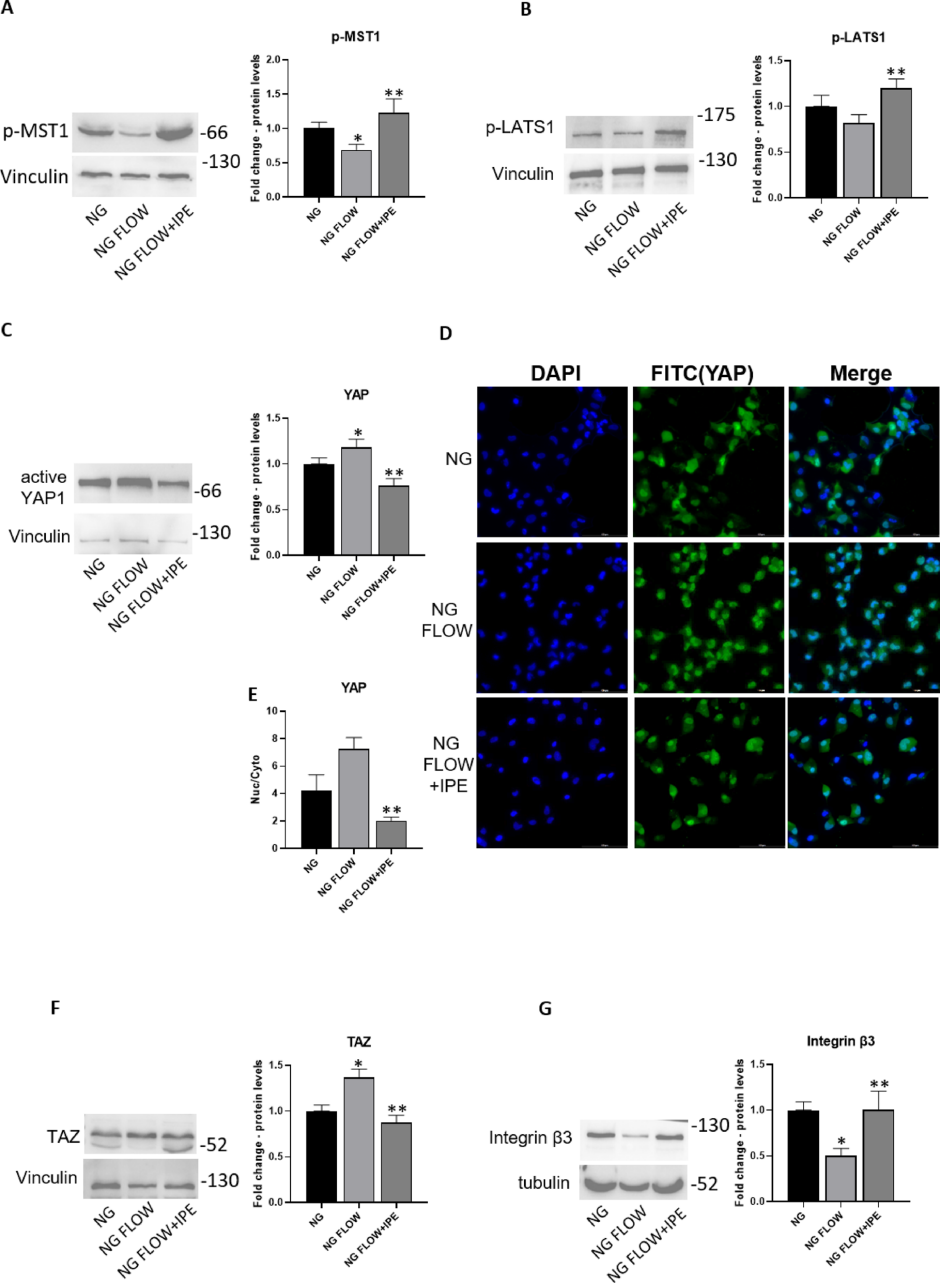
Effects of IPE on mechano-transduction in a dynamic shear stress model of cardiomyocytes. Western blot for p-MST1 (**A**), p-LATS1 (**B**), YAP (**C**), TAZ (**F**), Integrin β3 (**G**), in cardiomyocytes AC16 in static normal glucose condition (NG), cells exposed to turbulent flow in normal glucose condition (NG FLOW) and cells exposed to turbulent flow in normal glucose condition in presence of IPE (NG FLOW + IPE). The histograms show the densitometric analysis of 3 separate experiments representing the relative expression; NG value was set as 1. * *P* < 0.05 vs NG; ** *P* < 0.05 vs NG FLOW**. D** Immunofluorescence staining for active YAP (green) and nuclei (blue) in Ac16 in NG, NG FLOW, NG FLOW + IPE. Merge columns images show overlapping signals. Scale bar = 100 µm; E) YAP intensity ratio (nuclear/cytoplasmic) from cells randomly selected from 3 independent experiments. *P < 0.05 vs. NG; ** *P* < 0.05 vs NG FLOW

Turbulent flow induced a significant reduction of protein expression levels for p-MST1 (0.69-fold change vs NG; *p* < 0.05), and a modest reduction for p-LATS1 in AC16 cell line cultured in normal glucose condition. The addition of IPE prevented such flow-mediated effects (1.78-fold change vs NG FLOW; *p* < 0.05; 1.46-fold change vs NG FLOW; *p* < 0.05) (Fig. 6A–B).

Shear stress exposure induced a significant up-regulation of YAP protein expression levels (1.2-fold change vs NG; *p* < 0.05); such negative-shear stress mediated impacts on YAP protein level/activity was antagonized by IPE treatment (0.58-fold change vs NG FLOW; *p* < 0.05) (Fig. 6C). Immunofluorescence staining revealed that exposure to turbulent flow induces activation and nuclear localization of YAP in AC16 cells; this effect was decreased by IPE treatment (Fig. 6D). Quantitative analysis of YAP intensity staining confirmed the shear stress-induced nuclear localization of YAP, an effect that was significantly attenuated by IPE treatment under the same conditions (0.28-fold change vs NG FLOW; *p* < 0.05) (Fig. 6E). Turbulent flow also induced a significant increase of protein expression levels for TAZ in AC16 cell line cultured in normal glucose condition (1.36-fold change vs NG; *p* < 0.05); the addition of IPE prevented such flow-mediated effect (0.64-fold change vs NG FLOW; *p* < 0.05) (Fig. 6F).

Shear stress exposure also induced a significant reduction of protein expression levels for Integrin β3, a kinase upstream to YAP/TAZ that could be activated by mechanical signals [33] [34], in Ac16 cardiomyocytes (0.5-fold change vs NG; *p* < 0.05). This flow-driven effect was prevented through co-treatment with IPE (twofold change vs NG FLOW; *p* < 0.05) (Fig. 6G).

### Effects of IPE on inflammation and oxidative stress in cardiomyocytes exposed to turbulemt flow

The established dynamic shear stress model of cardiomyocytes was used also to investigate the effects of IPE on inflammation and oxidative stress.

**Fig. 7:**
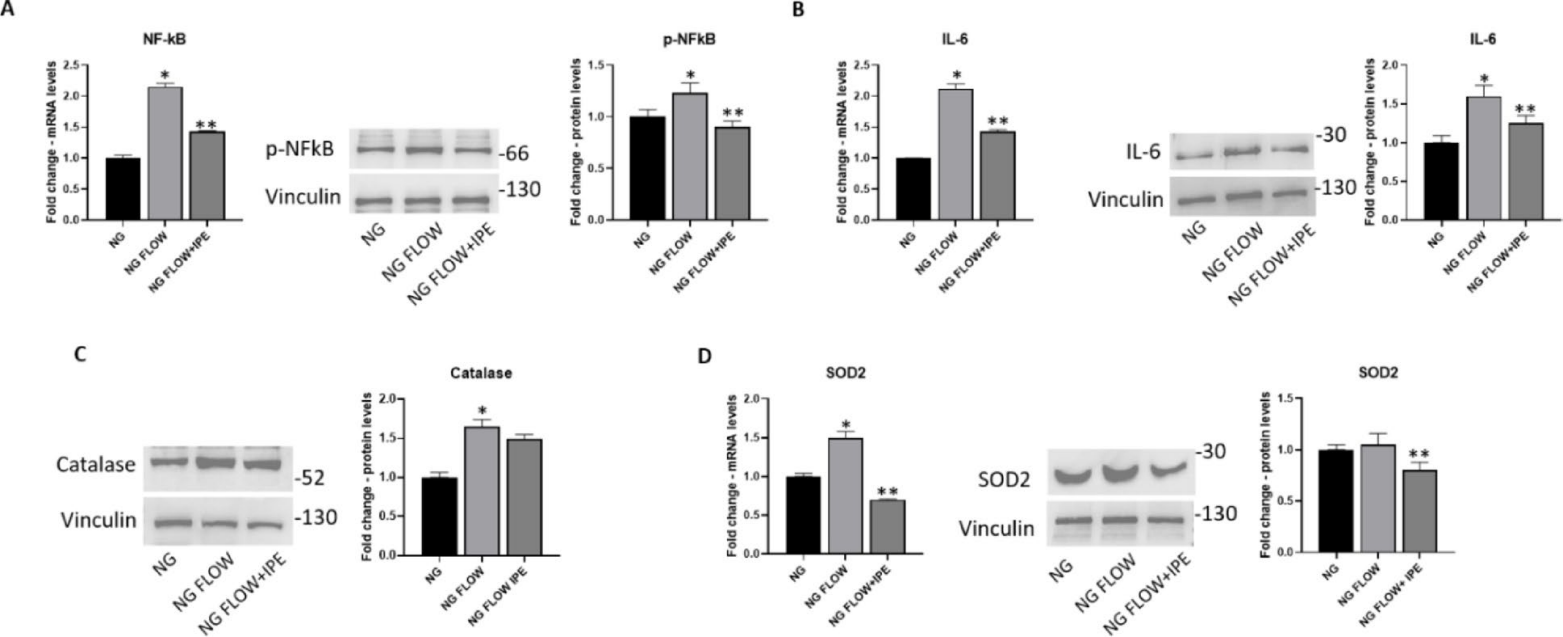
IPE effects on inflammation and oxidative stress in a dynamic shear stress model of cardiomyocytes. **A**, **B**, **E** qRT-PCR for NF-kB (**A**, left panel), IL-6 (**B**, left panel), SOD2 (**E**, left panel) in AC16 cells exposed to NG, NG FLOW, NG FLOW + IPE. β-Actin was used as internal control. The fold increase of mRNA expression compared with NG was calculated using the 2^−ΔΔCt^ method. Data are mean ± SEM. * *P* < 0.05 vs NG; ** *P* < 0.05 vs NG FLOW. Western blot for p-NF-kB (**A**, right panel), IL-6 (**B**, right panel), Catalase (**D**), SOD2 (**E**, right panel) in cardiomyocytes AC16 in NG, NG FLOW, NG FLOW + IPE. The histograms show the densitometric analysis of 3 separate experiments representing the relative expression; NG value was set as 1. * *P* < 0.05 vs NG; ** *P* < 0.05 vs NG FLOW

Turbulent flow induced a significant up-regulation of mRNA of NF-kB (2.15-fold change to NG; *p* < 0.05), and p-NF-kB protein levels (1.23-fold change to NG; *p* < 0.05) (Fig. 7A), as well as a significant increase of RNA and protein expression levels for IL-6 (1,6 -fold change to NG; *p* < 0.05) (Fig. 7B); all markers are significantly reduced by IPE treatment (NF-kB: 0.66-fold change vs NG FLOW; *p* < 0.05; p-NF-kB: 0,67-fold change vs NG FLOW; *p* < 0.05; IL-6: 0,78-fold change vs NG FLOW; *p* < 0.05) (Fig. 7A–B).

Turbulent flow also induced a significant increase in mRNA for other inflammatory markers, such as MCP-1 and VCAM-1, a phenomenon contrasted by IPE (Supplementary Fig. 6).

Regarding oxidative stress, exposure to turbulent flow induced overexpression of Catalase and SOD2, both reduced by IPE treatment, SOD2 in a statical manner (0, 57-fold change vs NG FLOW; *p* < 0.05) (Fig. 7 C–D).

### Effects of IPE on metabolism in cardiomyocytes in response to shear stress

**Fig. 8:**
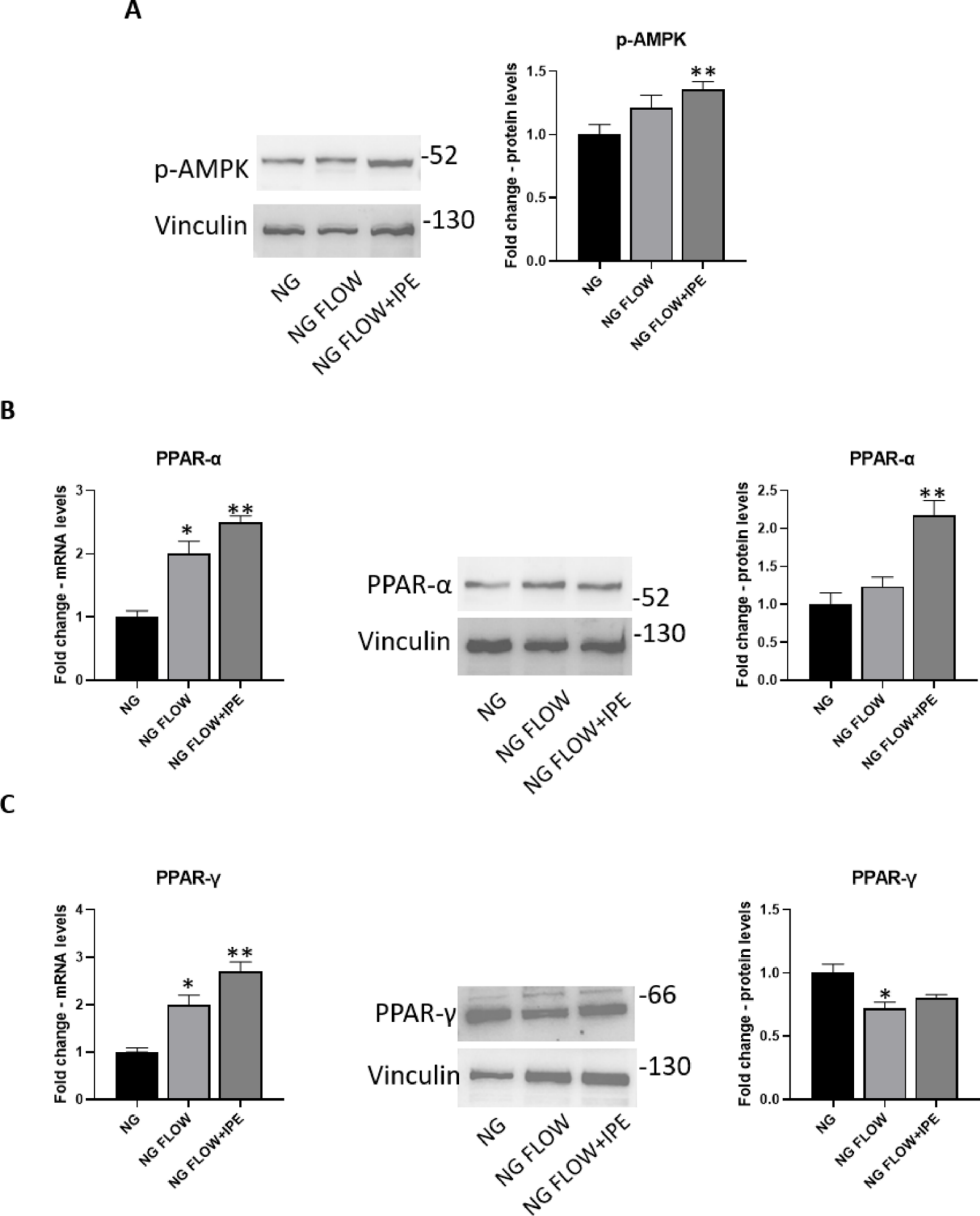
IPE effects on metabolism in a dynamic model of shear stress of cardiomyocytes. **A** Western blot for p-AMPK in AC16 exposed to NG, NG FLOW, NG FLOW + IPE. B-C) qRT-PCR and Western blot for PPAR-α (**B**) and PPAR-γ (**C**) in AC16 exposed under NG, NG FLOW, NG FLOW + IPE conditions. For qRT-PCR, β-Actin was used as internal control. The fold increase of mRNA expression compared with NG was calculated using the 2^−ΔΔCt^ method. Data are mean ± SEM. * *P* < 0.05 vs NG; ** *P* < 0.05 vs NG FLOW. For Western blot analysis, the histograms show the densitometric analysis of 3 separate experiments representing the relative expression; NG value was set as 1. * *P* < 0.05 vs NG; ** *P* < 0.05 vs NG FLOW

Turbulent flow exposure induced an increase of protein expression levels for p-AMPK and mRNA levels for PPAR-α (twofold change vs NG; *p* < 0.05) in AC16; these effects were further amplified in presence of IPE (1, 25-fold change vs NG FLOW; *p* < 0.05) (Fig. 8A–B). Moreover, turbulent flow induced a significant reduction of protein expression levels of PPAR-γ (0,sevenfold change vs NG; *p* < 0.05), that was partially antagonized by IPE addition (Fig. 8C).

## Discussion

We explored the cardioprotective effect of IPE, a novel purified ethyl ester of omega-3 EPA, particularly focusing on mechano-transduction process. Firstly, a meta-analysis of clinical trials with omega 3-fatty acids demonstrated their beneficial effects on cardiac contraction and on inflammation in patients with cardiovascular and cardiometabolic diseases. Secondly, we focused on mechano-transduction, demonstrating that IPE can affect this process in an in vitro model of human cardiac AC16 cells exposed to high glucose. We further confirmed the effect of IPE on mechano-transduction process using a dynamic shear stress model that mimics in vivo flow conditions. A role for IPE in the control of genes involved in inflammation, oxidative stress, and metabolism was also observed in static as well as in dynamic model.

Omega-3 fatty acids (FAs), such as EPA and DHA, exerted cardio-protective effects. Randomized clinical trials have highlighted that dietary intake of omega-3 FAs improves the prognosis of patients with MI or heart failure [35]. A reduction of major coronary events (MACE) was also demonstrated in the JELIS study [36] and in REDUCE-IT trial [7].

Several mechanisms have been suggested to explain the cardioprotective effects of omega-3 FAs by modulating blood lipids, such as TG, LDL, and HDL, thereby reducing atherosclerosis risk [37]. Moreover, omega-3 FAs, through enhancement of endothelial function, by reducing inflammation, and decreasing oxidative stress, contribute to protect against the development of CVDs [38]. In particular, Icosapent ethyl (IPE), the ethyl ester of eicosapentaenoic acid (EPA), confers multiple cardiovascular benefits, including triglyceride reduction, modulation of inflammation through pro-resolving mediators, antioxidant activity, and stabilization of atherosclerotic plaques, thereby providing protective effects on cardiovascular cells that go beyond its lipid-lowering properties. [5, 6]. To date, no study has examined the effects of omega-3 fatty acids on mechano-transduction, a critical process linking cardiac contraction to intracellular signaling, converting mechanical stimuli in biochemical signal. YAP and TAZ, the main mediators of mechano-transduction, represent a central signalling node integrating both metabolic and mechanical stimuli relevant to cardiomyocyte dysfunction. Indeed hyperglycaemia and shear stress affect YAP/TAZ pathway, driving their nuclear translocation and promoting inflammation and atherosclerosis [13, 14].

Shear stress is a key regulator of the cardiac mechanical environment. Within the ventricles, the pulsatile flow of blood generates highly heterogeneous endocardial shear forces, which impose complex mechanical stimuli on the endocardial endothelium and subendocardial cardiomyocytes. These forces influence not only cardiac contractility and mechano-sensitive signaling but also the structural remodeling of the myocardium [17, 18]. Cardiomyocytes sense the mechanical cues through stretch-activated ion channels, integrin-mediated focal adhesion, cytoskeleton remodelling, and activation of mechanosensitive transcriptional regulation such as YAP/TAZ [39]. In addition, paracrine factors released by shear-stressed endocardial cells—such as nitric oxide, endothelin-1, and growth factors—modulate cardiomyocyte survival, growth, and adaptive responses [19, 20].

Alterations in vascular shear stress can have profound downstream effects on cardiac function. Microvascular dysfunction and damage to the endothelial glycocalyx can impair oxygen delivery, increase oxidative stress, and reduce nitric oxide bioavailability, triggering dysregulation of signaling pathways in cardiomyocytes, including MAPK, PI3K/Akt, and ROS-mediated responses [21]. Changes in vascular shear stress that impair oxygen delivery and endothelial signaling can indirectly affect cardiomyocyte function, especially in hyperglycaemia or microangiopathies, where endothelium–cardiomyocyte crosstalk is crucial [22, 23, 40].

Our first in vivo meta-analysis on omega-3 fatty acids showed small but clinically relevant increases in LVEF% in patients with CVDs, exceeding known echocardiographic variability (± 5%) [41]. This finding suggested enhanced contractility possibly linked to mechano-transduction, and supported the potential role of IPE, a specific omega-3 derivative, in modulating this process.

Our in vitro results confirmed the previously observed changes in YAP and TAZ in response to high glucose in AC16 cardiomyocytes and demonstrated for the first time that IPE is able to reduce YAP/TAZ activity through the regulation of upstream kinases such as p-MST1 and p-LATS1. Furthermore, using a shear stress model of cardiomyocytes as a model to study cellular responses to hemodynamic forces, we showed that IPE reverted the decrease of kinases upstream to YAP/TAZ, p-MST1, and integrin β3, involved in the activation of YAP/TAZ induced by disturbed flow [33, 34].

These findings suggested a novel protective role of IPE in modulating mechano-transduction-driven responses under metabolic and mechanical stress in vitro.

Emerging evidence showed that YAP can regulate NF-kB activity, which in turn can enhance IL-6 production, suggesting a critical role for YAP signal cascade in inflammation [42]. Randomised control trials highlighted that a mix of DHA and EPA reduces plasma inflammatory markers [43–46]. Preliminary studies also demonstrated that omega-3 FAs may have a dual role in modulating inflammation by both inhibiting pro-inflammatory pathways, such as NF-κB and the NLRP3 inflammasome, and activating anti-inflammatory signalling pathways, such as the transcriptional activation of PPAR α/γ [47].

Interestingly, our results demonstrated a protective anti-inflammatory effect of IPE by reducing NF-κB and IL-6 levels in AC16 exposed to HG. A similar anti-inflammatory effect of IPE was also observed in the dynamic shear stress model. Moreover, the in vitro anti-inflammatory effect of IPE is supported by our in vivo meta-analysis indicating that omega-3 fatty acids, in general, exert anti-inflammatory effects, lowering IL-6, CRP and TNF-α expression in patients with cardiometabolic diseases.

Our findings also revealed that IPE plays a critical role in modulating PPAR-α and PPAR-γ, involved in inflammation control and in glucose and lipid metabolism [48]. Along with hyperglycaemia, IPE promoted an up-regulation of both receptors, exerting a regulatory effect on metabolic and anti-inflammatory pathways, a result also observed under shear stress in vitro conditions.

Additionally, our results demonstrated the ability of IPE to induce activation of p-AMPK, a key enzyme in cellular energy homeostasis [49]. This is particularly relevant in the context of diabetic cardiomyopathy, where AMPK (AMP‑activated protein kinase) functions as a central metabolic sensor regulating energy balance, mitochondrial function, and oxidative stress. Under hyperglycemic conditions, AMPK activity is often suppressed, contributing to cardiac dysfunction [50, 51]. Therefore, the ability of IPE to enhance AMPK activation may represent a possible mechanism by which it exerts cardioprotective effects, linking its antioxidant and anti-inflammatory actions to the restoration of cellular homeostasis in metabolically stressed cardiomyocytes.

In the dynamic model, IPE amplified the activation of p-AMPK induced by turbulent flow, enhancing cellular adaptive responses to mechanical stress.

Several lines of evidence indicated a beneficial effect for DHA and EPA through a decrease of ROS production, although contrasting results on the effect of these FAs on the expression of anti-oxidant molecules were published [52, 53]. Interestingly, our results demonstrated that IPE exerted an antioxidant effect by reducing high glucose-ROS production, lowering oxidative stress and consequently reducing the compensatory upregulation of endogenous antioxidant enzymes catalase and SOD2. Consistently, in the dynamic shear stress model, IPE was able to reduce protein expression levels of catalase and SOD2, confirming its beneficial effect against turbulent flow-induced oxidative stress in vitro.

Clinical observations and in vitro studies also demonstrated the protective effects of omega-3 FAs against apoptosis and cell death [37]. Our findings showed a modulation exerted by IPE for Bcl-2 protein, despite the difference between Bcl-2 mRNA and protein levels that probably reflects post-transcriptional and post-translational regulation, and supported the overall anti-apoptotic role of IPE, given by a marked reduction of the BAX/Bcl-2 ratio, a critical apoptotic prognostic marker significantly increased by HG exposure.

## Conclusions

Our results demonstrated a potential role of IPE in modulating the activation of mechano-transduction process in response to hyperglycaemia in vitro, through regulation of YAP/TAZ signalling pathway, resulting in a reduction of inflammation and oxidative stress. A cardioprotective mechanism of IPE, through modulation of apoptosis and metabolism, was also demonstrated. These findings could be relevant in diabetes, where hyperglycaemia leads to aberrant mechano-transduction process, sustained inflammation, and oxidative stress, all contributing to disease progression. In addition, our findings on a simplified shear-stress model allowed us to investigate cellular responses under mechanical loading. The effect of IPE on YAP/TAZ, that mitigates the disturbed flow-induced activation of pro-inflammatory pathways and oxidative stress, supported the cardioprotective role of IPE and suggested new potential therapeutic strategies for the treatment of cardiovascular disorders associated with disturbed blood flow and hemodynamic stress.

## Limitations

We acknowledge that our study has several limitations. First, our meta-analysis did not separately evaluate IPE and other omega-3 formulations with respect to LVEF and CVD outcomes, partly due to the limited availability of sufficiently powered trials for such comparisons. Nevertheless, it provides a comprehensive overview of the cardioprotective effects of omega-3 in general, supporting our in vitro investigations. Second, our analyses were performed exclusively in vitro, and future in vivo experiments are required to validate and extend these findings. As an alternative to in vivo experiments, we performed ex vivo meta-analyses at least in part the physiological relevance of our study.

Third, while DCFDA was used to assess intracellular ROS, this probe is non-specific and cannot distinguish among individual ROS; however, it provides a reliable measure of overall oxidative stress, which was the focus of our study.

Moreover, some molecules were assessed only by a single method, which may limit cross-validation of the study. However, Western blot appeared to us the most feasible and reliable approach, allowing semi-quantitative detection of the activation of key signaling mediators in mechanotransduction in high-glucose-stimulated cardiomyocytes.

## Supplementary Information

Below is the link to the electronic supplementary material.

Supplementary Material 1.

## Data Availability

The data used and/or analyzed during the current study are available from the corresponding author on reasonable request.

## Abbreviations

FA: Fatty acids
IPE: Icosapent ethyl
EPA: Eicosapentaenoic acid
YAP: Yes-associated protein
TAZ: Transcriptional co-activator with PDZ-binding motif
NG: Normal glucose
HG: High glucose
LVEF: Left ventricular ejection fraction
HF: Heart failure
CVDs: Cardiovascular diseases
T2DM: Type 2 diabetes mellitus
TNF-α: Tumour Necrosis Factor alpha
IL-6: Interleukin 6
CRP: C-reactive protein
MI: Myocardial infarction
p-MST1: Phospho-mammalian sterile 20-like kinases 1
p-LATS1: Phospho-large tumor suppressor kinase 1
NF-kB: Nuclear Factor-kappa B
p-NF-kB: Phospho-nuclear factor-kappa B
ROS: Reactive oxygen species
SOD2: Superoxide dismutase 2
p-AMPK: Phospho-AMP-activated protein kinase
PPAR-α: Peroxisome proliferator-activated receptor gamma alpha
PPAR-γ: Peroxisome proliferator-activated receptor gamma
MCP-1: Monocyte chemoattractant protein-1
VCAM-1: Vascular cell adhesion protein 1
DHA: Docosahexaenoic acid
MACE: Major adverse cardiovascular events
TG: Tryglycerides
LDL: Low density lipoproteins
HDL: High density lipoproteins
CVDs: Cardiovascular diseases
NLRP3: Nucleotide-binding domain, leucine-rich–containing family, pyrin domain–containing-3
