# Supplementary material for: Cardioprotective mechanism of ω-3 fatty acid icosapent ethyl (IPE) in cardiomyocytes: role in high glucose and shear stress-induced mechano-transduction dysregulation
Source: Cardiovasc Diabetol. 2025 Dec 13;25:14. doi: 10.1186/s12933-025-03033-8 (PMC12805760; doi:10.1186/s12933-025-03033-8)
Supplement: Supplementary file 1 — Supplementary Material 1. [file 12933_2025_3033_MOESM1_ESM.docx]

**Cardioprotective mechanism of ω-3 fatty acid icosapent ethyl (IPE) in cardiomyocytes: role in high glucose and shear stress-induced mechano-transduction dysregulation**

Ada Pesapane^a^, Lucia Scisciola^a^, Manuela Giovanna Basilicata^a^, Rosaria Anna Fontanella^a^, Nunzia Balzano^a^, Annalisa Capuano^b,c^, Asad Zia^a^, Maryam Arshad^a^, Zeeshan Ulfat^a^, Giovanni Tortorella^a^, Ludovica Vittoria Marfella^a^, Alberta Maria Maddalena Palazzo^a^, Giuseppe Signoriello^d^, Celestino Sardu^a^, Giuseppe Paolisso^a,e^, Michelangela Barbieri^a^

***^a^*** *Department of Advanced Medical and Surgical Sciences, University of Campania "Luigi Vanvitelli", Naples, Italy*

***^b^*** *Department of Experimental Medicine, University of Campania "Luigi Vanvitelli", Naples, Italy*

*^c^* *Campania Regional Centre for Pharmacovigilance and Pharmacoepidemiology, Naples, Italy.*

*^d^Department of Mental and Physical Health and Preventive Medicine, Section of Medical Statistics, University of Campania "Luigi Vanvitelli", Naples, Italy*

***^e^*** *UniCamillus, International Medical University, Rome – Italy*

**Address correspondence to:**

Lucia Scisciola, Ph.D.

University of Campania “Luigi Vanvitelli”

Department of Advanced Medical and Surgical Sciences

P.zza L. Miraglia, 2

80138 Napoli

Tel: +390815665110

e-mail: [lucia.scisciola@unicampania.it](mailto:lucia.scisciola@unicampania.it)

**Additional File**

**Supplementary Figure 1**

AC16 cells were perfused in the LiveBox1 chamber (Ivtech) at flow rates of 500 µL/min. Turbulence is a threshold phenomenon: once it sets in, characteristic vortices form, where the mechanical stress increases in an uncontrolled manner. The increase in flow, combined with the chamber configuration, leads to a turbulent flow regime above a certain flow rate (e.g., 300 µL/min and above).

The shear stress has been evaluated, using a fluid dynamic computational model (CFD), basing on the sofware SimFlow. the shear stress values exhibit a wide range, making it inappropriate to represent the chamber’s shear stress with a single value, as this would lack statistical significance. By analyzing the shear stress along a direction perpendicular to the liquid flow (e.g., the inlet-to-outlet axis in the LB1 chamber), the distribution presented in the graph can be obtained. This provides a more detailed interpretation of the information conveyed by the colored map. The map illustrates the shear stress distribution at the bottom of a LiveBox chamber under a flow rate of 500 μL/min, with values expressed in Pa. The maximum shear stress is observed in the central region of the glass, corresponding to the red area on the map, while the values gradually decrease toward the edges. Based on these observations, we consider it unnecessary to convert the units to dyne/cm². The results are summarized in the distribution graph (A) and the coloured map (B) represented in the figure.

**
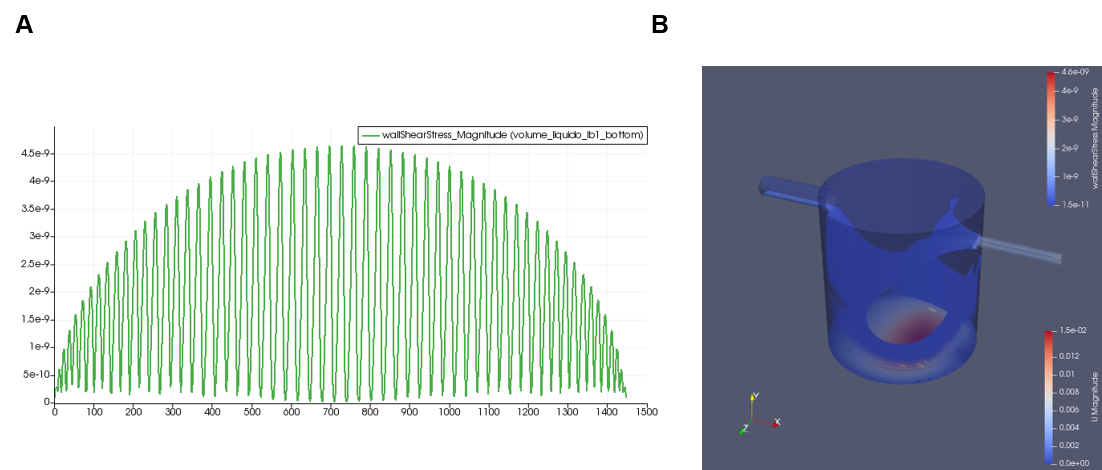
**

**Supplementary Figure 2**

A PRISMA flow chart summarized the study selection process for the meta-analysis investigating left ventricular ejection fraction (LVEF%) in CVD patients treated with omega-3 fatty acids or placebo. After removing duplicates, titles and abstracts were screened to remove reviews, editorial/letter to editor, studies with different outcomes or different populations. Full-text articles assessed for eligibility were subsequently examined in detail to evaluate study design, population characteristics, intervention type, and available outcomes. Studies not meeting the criteria were excluded, with reasons documented at each step. The final meta-analysis included three studies.


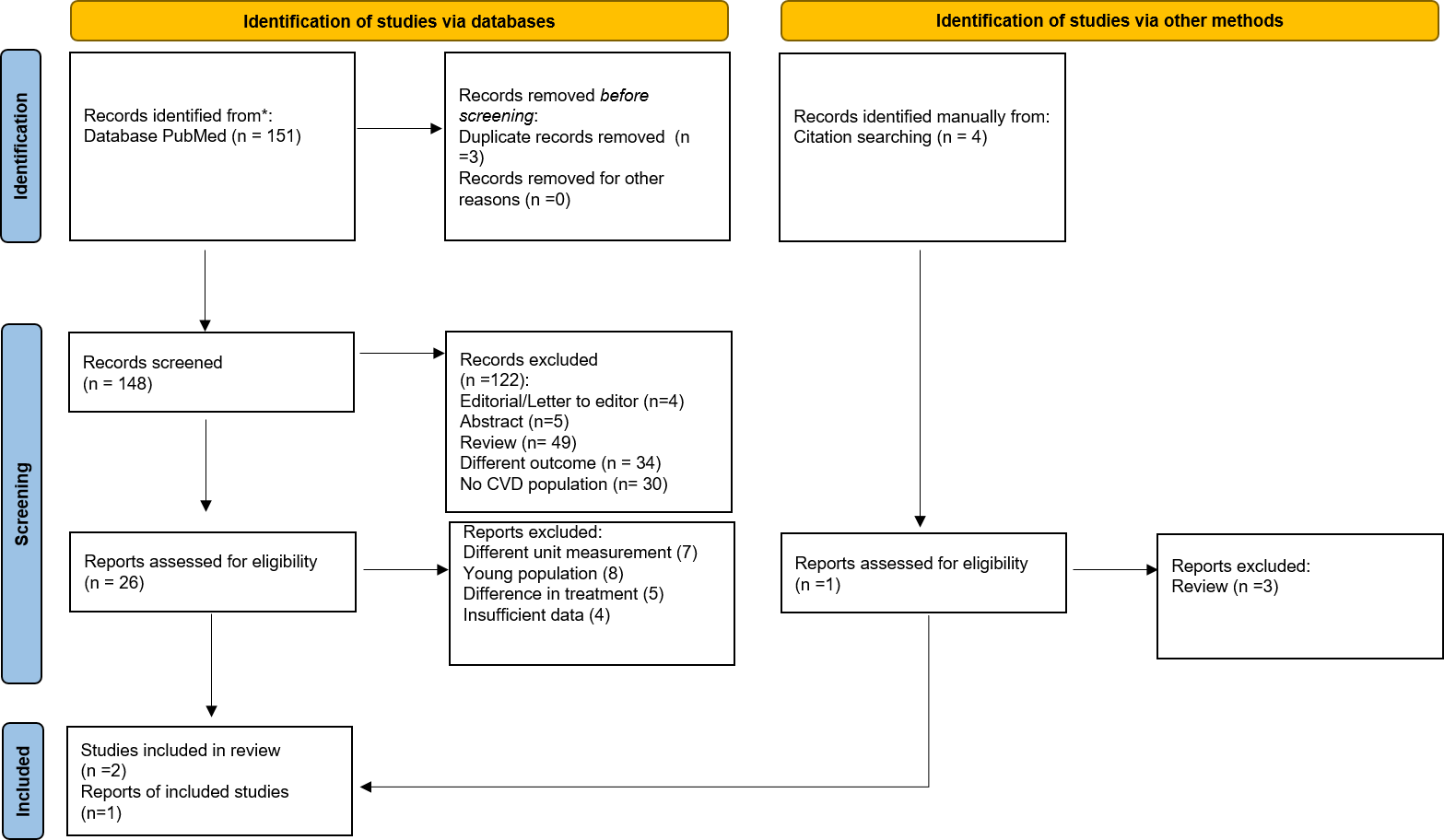


**Supplementary Figure 3**

A PRISMA flow chart summarized the study selection process for the meta-analysis investigating inflammation markers (IL-6, TNF-α, CRP) in patients with cardiometabolic diseases (HF, CVDs and T2DM) treated with omega-3 fatty acids or placebo was prepared. After removing duplicates, titles and abstracts were screened to remove reviews, editorial/letter to editor, studies with different outcomes or different populations. Full-text articles assessed for eligibility were subsequently examined in detail to evaluate study design, population characteristics, intervention type, and available outcomes. Studies not meeting the criteria were excluded, with reasons documented at each step. The final meta-analysis included a total of 11 studies: two for CVDs, three for T2DM and six for HF.


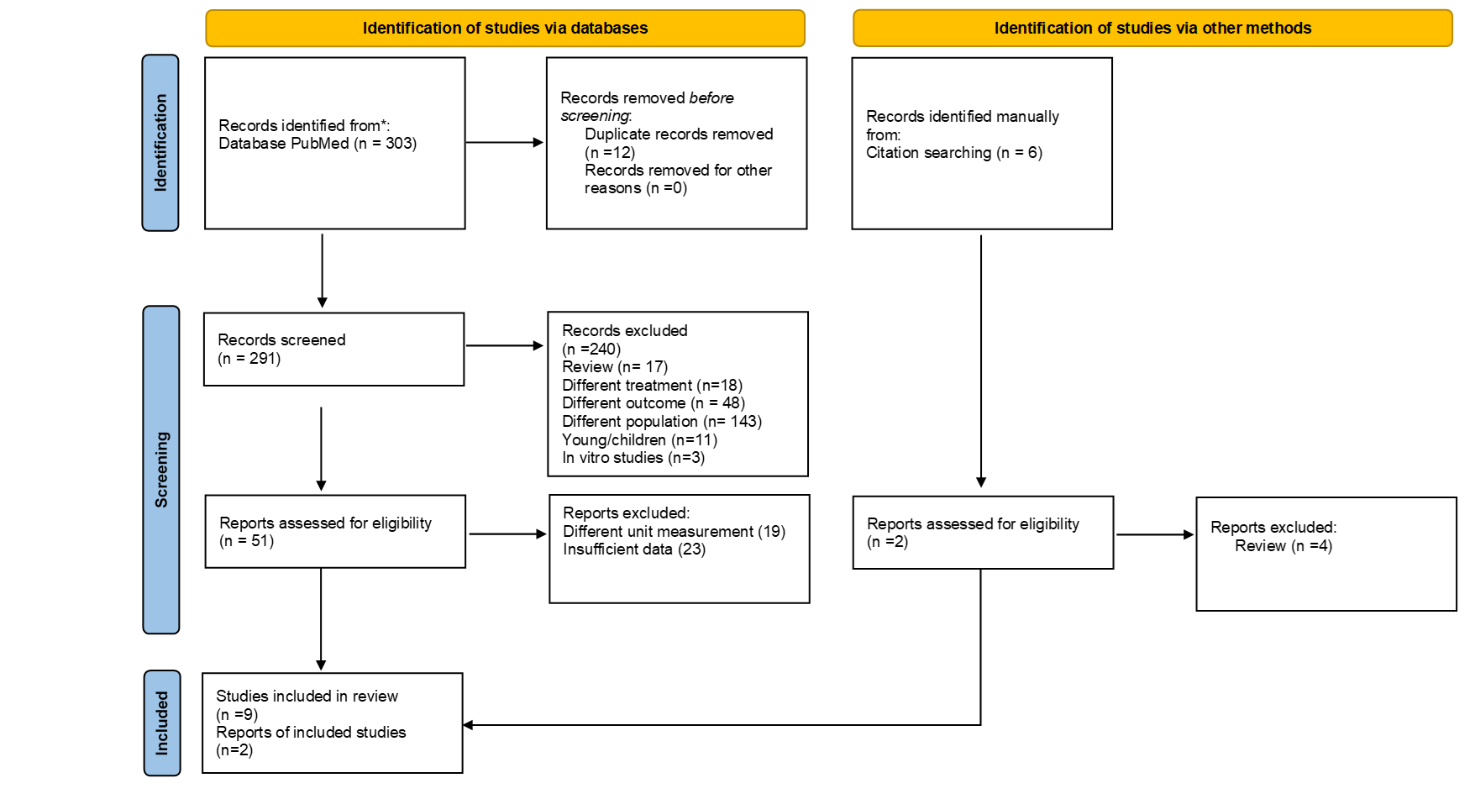


**Supplementary Figure 4**

The influence of each individual study on the overall effect of the meta-analysis on inflammation markers (IL-6, TNF-α, CRP) in patients with cardiometabolic diseases treated with omega-3 fatty acids was evaluated by a sensitivity analysis (Leave-one-out analysis) performed using Stata 18.

Regarding the TNF-α endpoint, leave-one-out variations are minimal, indicating robust evidence. Although heterogeneity (I²) remains high across iterations, reflecting intrinsic variability among studies, it does not compromise the stability of the overall estimate. For the IL-6 endpoint, the overall estimate remains relatively stable in the leave-one-out analysis. Although heterogeneity is still high—reflecting methodological differences—it does not undermine the robustness of the results. Differently, the CRP subgroup shows the greatest instability: in some iterations, excluding individual studies leads to marked shifts in the pooled effect. Residual heterogeneity (I²) is very high and often increases in the leave-one-out analysis. One or two studies appear to exert substantial influence (outliers or high-leverage points). Overall, CRP provides the least robust evidence among the analyzed endpoints. The pronounced heterogeneity suggests major differences across studies (patient characteristics, timing of measurements, laboratory methods). To address this, an analysis excluding the Pooya 2010 study was performed: heterogeneity improved, and the overall between-group differences remained non-significant.

**Supplementary Figure 5**

A dose-curve response was used to define the concentration of IPE to carry out experiments. Cell viability was assayed by Cell Counting Kit-8 (CCK-8, CK04, Dojindo) according to the manufacturer's protocols. Briefly, AC16 cells were seeded into 96-well plates and treated with IPE (ranging from 10 µM to 50 µM) for the two time points used in our study—72 hours and 7 days. After specific treatment, 10 μL of CCK-8 solution was added to each well and incubated for 2 h at 37°C. The absorbance was then recorded at 450 nm using a microplate reader (Infinite M Nano^+^ plate reader, TECAN). The relative cell viability was normalized with the control group (NG) using optical density values, and three independent experiments were conducted. No statistical differences were observed for cells treated with IPE 40 μM compared to control (NG) at 72hours (A) and at 7 days (B). Lower doses of IPE were ineffective in our setting.


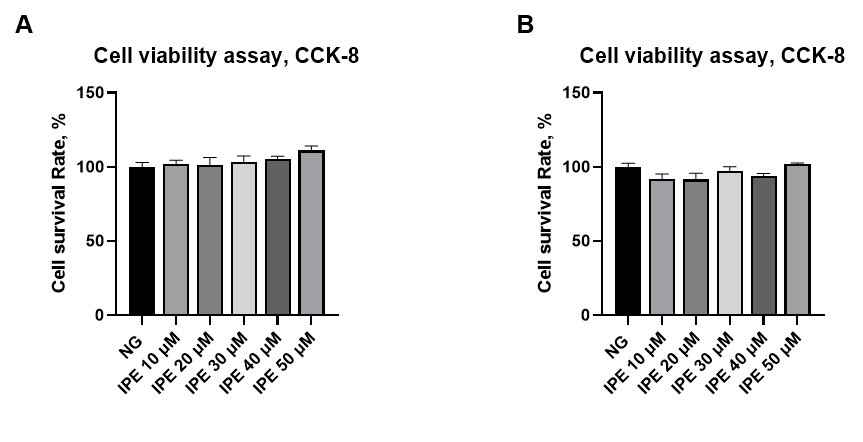


**Supplementary Figure 6**

mRNA expression level for the inflammatory markers MCP-1 and VCAM-1 was evaluated in AC16 cells exposed to NG, NG FLOW, NG FLOW + IPE, by qRT-PCR, using the following primers:

MCP-1: fw 5′-ACTGAAGCTCGTACTCTC-3′, rv 5′-CTTGGGTTGTGGAGTGAG-3′; VCAM-1: fw: 5’-AGTTGAAGGATGCGGGAGTAT-3’, rv: 5’-GGATGCAAAATAGAGCACGAG-3’; β-actin: fw 5′-CATCCGCAAAGACCTGTACG-3′, rv 5′-CCTGCTTGC TGATCCACATC-3′. β-Actin was used as internal control. The fold increase of mRNA expression compared with NG was calculated using the 2^−ΔΔCt^ method. The result showed that IPE reduce mRNA expression levels of MCP-1 (A) and VCAM-1 (B) in AC16 cells exposed to NG+FLOW.

Data are mean ± SEM. * P <0.05 vs NG; ** P <0.05 vs NG FLOW.

**
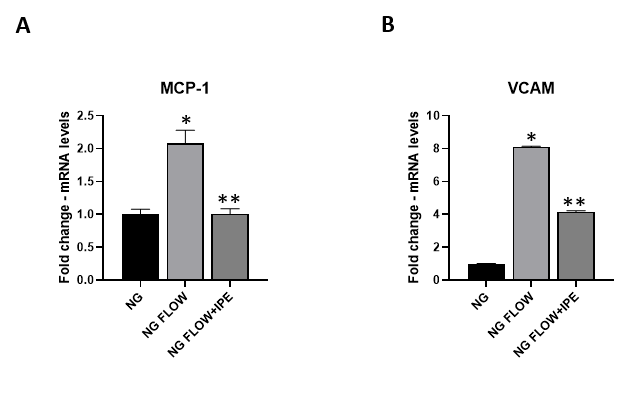
**
